# Estimated Glomerular Filtration Rate Is a Poor Predictor of the Concentration of Middle Molecular Weight Uremic Solutes in Chronic Kidney Disease

**DOI:** 10.1371/journal.pone.0044201

**Published:** 2012-08-31

**Authors:** Nathalie Neirynck, Sunny Eloot, Griet Glorieux, Daniela V. Barreto, Fellype C. Barreto, Sophie Liabeuf, Aurélie Lenglet, Horst D. Lemke, Ziad A. Massy, Raymond Vanholder

**Affiliations:** 1 Nephrology Section, Department of Internal Medicine, Ghent University Hospital, Gent, Belgium; 2 INSERM U-1088, Amiens, France; 3 Clinical Research Centre, Division of Clinical Pharmacology, Amiens University Hospital, Amiens, France, and the Jules Verne University of Picardy, Amiens, France; 4 Division of Nephrology, Amiens University Hospital, Amiens, France; 5 EXcorLab GmbH, Obernburg, Germany; University of Sao Paulo Medical School, Brazil

## Abstract

**Background:**

Uremic solute concentration increases as Glomerular Filtration Rate (GFR) declines. Weak associations were demonstrated between estimated GFR (eGFR) and the concentrations of several small water-soluble and protein-bound uremic solutes (MW<500Da). Since also middle molecular weight proteins have been associated with mortality and cardiovascular damage in Chronic Kidney Disease (CKD), we investigated the association between several eGFR formulae and the concentration of Low Molecular Weight Proteins (LMWP) (MW>500Da).

**Materials and Methods:**

In 95 CKD-patients (CKD-stage 2–5 not on dialysis), associations between different eGFR-formulae (creatinine, CystatinC-based or both) and the natural logarithm of the concentration of several LMWP’s were analyzed: i.e. parathyroid hormone (PTH), Cystatin C (CystC), interleukin-6 (IL-6), tumor necrosis factor-alpha (TNF-α), leptin, retinol binding protein (RbP), immunoglobin light chains kappa and lambda (Ig-κ and Ig-λ), beta-2-microglobulin (β_2_M), myoglobin and fibroblast growth factor-23 (FGF-23)).

**Results:**

The regression coefficients (R^2^) between eGFR, based on the CKD-EPI-Crea-CystC-formula as reference, and the examined LMWP’s could be divided into three groups. Most of the LMWP’s associated weakly (R^2^ <0.2) (FGF-23, leptin, IL-6, TNF-α, Ig-κ, Ig-λ) or intermediately (R^2^ 0.2–0.7) (RbP, myoglobin, PTH). Only β_2_M and CystC showed a strong association (R^2^ >0.7). Almost identical R^2^-values were found per LMWP for all eGFR-formulae, with exception of CystC and β_2_M which showed weaker associations with creatinine-based than with CystC-based eGFR.

**Conclusion:**

The association between eGFR and the concentration of several LMWP’s is inconsistent, with in general low R^2^-values. Thus, the use of eGFR to evaluate kidney function does not reflect the concentration of several LMWP’s with proven toxic impact in CKD.

## Introduction

Chronic Kidney Disease (CKD) is an independent risk factor for mortality and cardiovascular disease (CVD) [1]. As Framingham risk calculation cannot correctly predict this risk [2], [3], other than traditional risk factors are at play. When kidney function declines, retention of uremic solutes with potential to cause vessel damage and other toxic effects, conceivably plays a role in this [4], [5].

Glomerular Filtration Rate (GFR) is used to express kidney function and this can accurately be measured by time-consuming and labor-intensive methods [6]. In clinical practice, serum creatinine (Crea) based formulae are used to calculate estimated GFR (eGFR), which offer an acceptable estimate of measured GFR (mGFR) [6]–[9]. However, if possible, mGFR is to be preferred as it may differ from eGRF especially in the lower GFR range in a CKD population or in patients with a body constitution that deviates from the average [6], [10]. On the other hand, measuring GFR by one of these techniques is more costly and labor-intensive than to determine eGFR. Also, current guidelines classify CKD based on the Modification of Diet in Renal Disease study (MDRD) formula [11], [12]. More recently, the CKD-EPI-Crea formula [13] has been proposed as a valid alternative, especially if eGFR is >60 ml/min/1.73 m^2^ [9], so that it possibly will be incorporated into the upcoming KDIGO guideline [14].

Since concentrations of uremic solutes rise when GFR deteriorates, it has been thought that GFR reflects the retention state of the patient and that the elevation of individual solute concentration of uremic toxins is closely related to the gradual deterioration of GFR. However, Eloot *et al*. [15] found very low regression coefficients between eGFR and several low molecular weight retention solutes in a CKD population.

The low molecular weight proteins (LMWP) are among the main representatives of the middle molecules, the third family of uremic retention solutes [16], and are interesting to study for their relationship with eGFR as with normal kidney function they are freely filtered through the glomerular basement membrane (GBM) and then mainly degraded into amino acids by the proximal tubules [17]. Furthermore the concentrations of several of the investigated LMWP’s, such as inflammatory parameters and FGF-23, are already elevated in patients with a moderate reduction in GFR [18]–[21] or in more advanced CKD [22]. As a consequence, associations between these solutes and eGFR are often assumed. Assessing the predictive value of eGFR for their concentration is furthermore also relevant, because several LMWP’s, such as interleukin-6 (IL-6) [23]–[26], tumor necrosis factor-alpha (TNF-α) [27], [28], beta-2-microglobulin (β_2_M) [29], [30], and fibroblast growth factor-23 (FGF-23) [31]–[33], have been linked to mortality or surrogate outcomes like vascular damage or progression of kidney failure. In addition, active removal of middle molecules by dialysis has been associated with better outcome [34].

Therefore, we investigated in a CKD population whether the concentration of several LMWP’s would associate with eGFR, calculated by several eGFR formulae.

## Materials and Methods

### Ethics Statement

The study was approved by the local ethical committee (Comité Consultatif de Protection des Personnes dans la Recherche Biomédicale (CCPPRB) de Picardie, CHU Amiens, Amiens, France) and performed in accordance to the Declaration of Helsinki. Written informed consent was obtained from all patients.

### Study Population

This evaluation is a planned sub-analysis of a study undertaken over an 18-month period (January ’06- June ’07), which screened 150 Caucasian patients with prevalent CKD stage 2–5D from the Nephrology Department at Amiens University Hospital, in which uremic retention solutes in relation to clinical outcomes were analyzed [23], [35]–[37].

All patients were over 40 years old and had a confirmed diagnosis of CKD (two previous eGFR measures of <90 ml/min, calculated by the Cockcroft-Gault formula with an interval of 6–9 months) [38]. Exclusion criteria were chronic inflammatory disease, atrial fibrillation, complete heart block, abdominal aorta aneurysm, an aortic and/or femoral artery prosthesis, primary hyperparathyroidism, kidney transplantation, and any acute cardiovascular event in a 3 month period prior to screening for inclusion.

From the 140 patients who met the inclusion criteria, 45 were excluded from the current study because of hemodialysis treatment, which has an impact on solute concentration and on eGFR. The 95 patients, included in this study, were classified in CKD stages according to the CKD-EPI-Crea-CystC formula for further analysis [8].

### Sampling and Laboratory Methods

Blood samples of all patients were collected in the morning from 9 a.m. on, centrifuged, aliquoted, frozen and stored at −80°C. Cystatin C (CystC) (MW: 13.3 kDa) concentration was determined by immune-nephelometry (N latex Cystatin C®, Siemens Healthcare, Dade Behring, Marburg, Germany) and that of intact parathyroid hormone (PTH) (MW: 9.5kDa) with a chemiluminometric immunoassay (Liaison N-tact PTH CLIA®, Diasorin, Stillwater, MN, USA). The determination of retinol binding protein (RbP) (MW: 21kDa), beta-2-microglobulin (β_2_M) (MW: 11.8kDa), myoglobin (MW: 17kDa) and total immunoglobulin light chains kappa (Ig-κ) and lambda (Ig-λ) (MW: 23kDa) was performed by laser nephelometry (BNProSpec®, Siemens Healthcare, Dade Behring, Marburg, Germany). ELISA’s were used to determine the levels of interleukin-6 (IL-6) (MW: 23kDa), tumor necrosis factor-alpha (TNF-α) (MW: 17kDa) (R&D Systems, Wiesbaden, Germany), and leptin (MW: 16kDa) (DRG diagnostics, Marburg, Germany). Intact fibroblast growth factor-23 (FGF-23) (MW: 32kDa), was measured by a two-site (N-terminal and C-terminal) ELISA (Immunotopics, San Clemente, CA, USA). Serum creatinine (Crea) (MW: 113Da) was measured colorimetrically by standard laboratory methods.

### eGFR- Calculation

Six different formulae were used to estimate GFR: the CKD-EPI formula, based on Crea and CystC (CKD-EPI-Crea-CystC) eGFR = 177.6·Crea^−0.65^·CystC^− 0.57^·age^−0.20^·0.82 (if female) [8]; two formulae based on Crea alone: the MDRD eGFR = 175·Crea^−1.154^·age^−0.203^·(0.742 if female)·(1.21 if black) [7] and the CKD-EPI creatinine (CKD-EPI-Crea) eGFR = 141.min(Crea/κ,1)^α^·max(Crea/κ,1) ^−1.209^·0.993^Age^·1.018 (if female)·1.159 (if black) (κ: 0.7 if female, 0.9 if male; α: −0.329 if female, −0.411 if male) [13]; and three formulae based on CystC alone: Stevens eGFR = 127.7·CystC^−1.17^·age^−0.13^·0.91 (if female)·1.06 (if black) [8], Le Bricon eGFR = [78·(1/CystC)]+4 [39] and Rule eGFR = 66.8·(CystC)^−1.3^ [40].

### Statistical Analysis

The data are expressed as mean ± standard deviation and analysed by ANOVA if they were normally distributed. For data that were not normally distributed, median with interquartile range and Kruskall-Wallis test were used. Linear regressions and Pearson correlations were calculated on semi-logarithmic (LN) concentrations as a function of eGFR. Multifactorial analysis was performed to correct for well-known influencing factors for the concentration of several solutes. The regression model of CystC, β_2_M, IL-6, TNF-α, Ig-κ and Ig-λ was adjusted for C-reactive protein (CRP), the one of FGF-23 and PTH for calcium, phosphorus and vitamin D-supplementation, the one of leptin for body mass index (BMI) and gender, and the one of RbP for BMI, 1/CRP and diabetes mellitus. A P<0.05 was considered as statistically significant. All statistical analyses were performed using SPSS Statistics 19 (SPSS Inc, Chicago, IL) for Windows (Microsoft Corp, Redmond, WA).

## Results

Ninety-five patients at different stages of CKD were included: 11.5% CKD stage 2, 39.0% CKD stage 3, 39.0% CKD stage 4, and 10.5% CKD stage 5 not on dialysis. Table 1 summarizes the demographic and clinical characteristics of the study population.

**Table 1:** Main demographic and clinical characteristics of the study population (n = 95).

	CKD stage					P
	Stage 2–5	stage 2	stage 3	stage 4	stage 5	
Number n (%)	95 (100)	11 (11.5)	37 (39.0)	37 (39.0)	10 (10.5)	
eGFR (ml/min/1.73m^2^)	35±18	69±8	43±9	22±4	11±3	<0.001
Age (years)	68±12	65±8	69±12	65±13	66±15	0.07
Male gender n (%)	59 (62)	9 (82)	24 (65)	22 (60)	4 (40)	0.39
Diabetes Mellitus n (%)	45 (47)	4 (36)	19 (51)	18 (49)	4 (40)	0.50
BMI (kg/m^2^)	29±7	26±5	29±6	31±7	28±7	0.28
Cholesterol (mmol/l)	5.0±1.1	5.4±0.7	4.6±1.1	5.3±1.1	4.6±0.5	0.02
Triglycerides (mmol/l)	1.9±1.4	1.7±0.9	1.6±0.7	2.4±1.9	2.2±1.2	0.06
CRP (mg/l)	3.11 [1.1–6.7]	2.3 [0.7–4.9]	2.8 [1.4–5.0]	3.7 [0.8–8.5]	4.1 [0.4–15.6]	0.696
Albumin (g/l)	38.9±6.4	40.6±8.8	38.4±5.7	39.9±5.8	33.8±6.7	0.07
Hemoglobin (g/l)	12.5±1.7	14.0±1.2	12.7±1.5	12.0±1.6	10.9±1.4	<0.001
Calcium (mmol/l)	2.3±0.1	2.3±0.1	2.3±0.1	2.3±0.2	2.3±0.2	0.96
Phosphate (mmol/l)	1.2±0.3	0.9±0.3	1.1±0.2	1.4±0.3	1.5±0.5	<0.001
Vit D supplement n (%)	17 (18)	1 (9)	5 (13)	6 (16)	5 (50)	0.06

CKD stages according to the CKD-EPI-Crea-CystC formula. Data are expressed as mean ± SD, median with interquartile range between square brackets or number for binary variables, with percentages between brackets per CKD class. CKD, chronic kidney disease; eGFR, estimated glomerular filtration rate; BMI, body mass index; Statistical analysis: ANOVA or Kruskall-Wallis; P-values comparing all stages.

The concentrations of the studied LMWP’s, except for the immunoglobulin light chains, increased progressively with declining kidney function (Table 2).

**Table 2:** Concentrations of uremic solutes ± standard deviation according to CKD-stage (CKD-EPI-Crea-CystC).

	CKD stage					P- value
	stage 2–5	stage 2	stage 3	stage 4	stage 5	
CystC (mg/l)	1.9±0.9	0.9±0.2	1.4±0.5	2.4±0.7	3.5±0.7	<0.001
β_2_M (mg/l)	6.1 [3.2–9.3]	2.6 [2.1–4.6]	3.8 [3.0–5.3]	8.2 [6.7–10.4]	13.9 [12.8–16.4]	<0.001
PTH (pg/ml)	77.0 [42.5–135.5]	39.5 [25.5–44.0]	62.0 [42.0–83.5]	124.0 [74.0–196.0]	111.5 [22.0–173.0]	<0.001
RbP (mg/l)	82.0±32.8	52.9±15.2	67.0±21.8	102.6±32.2	95.9±31.5	<0.001
Myoglobin (mg/l)	82.7 [54.9–115.0]	49.9 [32.4–57.4]	72.3 [49.7–104.8]	98.8 [75.4–125.5]	170.5 [62.8–244.0]	<0.001
IL-6 (pg/ml)	2.6 [1.3–5.1]	1.1 [0.4–1.9]	2.2 [1.3–4.0]	3.0 [1.3–5.2]	7.0 [2.2–14.0]	0.001
TNF-α (pg/ml)	3.4 [2.2–4.6]	1.1 [1.1–2.5]	4.1 [2.2–4.6]	2.6 [2.2–5.1]	4.1 [3.1–7.2]	0.016
Leptin (ng/ml)	12.8 [2.0–43.5]	0.56 [<0.48–5.3]	8.5 [2.5–33.4]	21.4 [4.3–59.2]	26.3 [0.7–>105]	0.012
FGF-23 (pg/ml)	30.6 [26.8–34.4]	26.6 [25.8–27.3]	31.5 [27.6–34.5]	30.6 [27.6–34.9]	34.4 [33.1–35.0]	0.007
Ig-κ (g/l)	2.6 [2.2–3.0]	2.4 [2.3–2.8]	2.4 [2.1–4.8]	2.8 [2.4–3.0]	2.5 [2.1–3.1]	0.330
Ig-λ (g/l)	1.5 [1.3–1.8]	1.5 [1.3–1.7]	1.5 [1.3–1.7]	1.6 [1.4–1.8]	1.2 [1.2–1.8]	0.280

Concentrations are expressed as mean ± SD or median with interquartile range (between square brackets) as appropriate. CKD: chronic kidney disease, CystC: cystatin C, β_2_M: beta-2-microglobulin, PTH: parathyroid hormone, RbP: retinol binding protein, IL-6: interleukin-6, TNF-α: tumor necrosis factor-alpha, Ig-κ: immunoglobulin light chain kappa, Ig-λ: immunoglobulin light chain lambda, FGF-23: fibroblast growth factor-23. Statistical analysis: ANOVA or Kruskall-Wallis; P comparing all stages.

**Figure 1:**
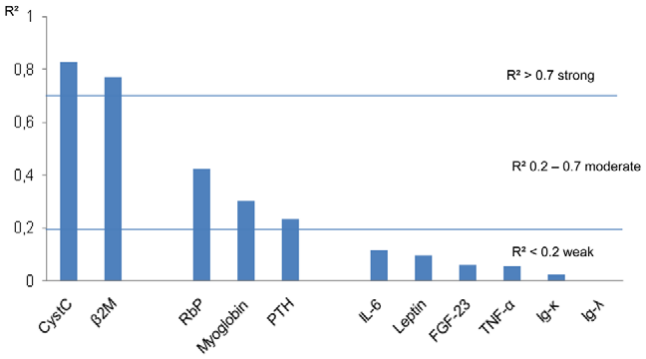
Regression coefficients between LN of studies LMWP’s and eGFR. The coefficients of the linear regression analysis between the natural logarithm of the studied low molecular weight protein concentrations and estimated Glomerular Filtration Rate, according to CKD-EPI-Crea-CystC, can be divided into 3 groups: strong (R^2^ >0.7), moderate (R^2^ 0.2–0.7) and weak (R^2^ <0.2). The dashed lines indicate R^2^ = 0.2 and 0.7. All correlations were significant except for Ig-κ and Ig-λ. LN: natural logarithm, LMWP: low molecular weight protein, eGFR estimated glomerular filtration rate, R^2^: regression coefficient, Cyst C: Cystatin C, β_2_M: beta-2-microglobulin, RbP: retinol binding protein, PTH: parathyroid hormone, IL-6: interleukin-6, FGF-23: fibroblast growth factor-23, TNF-α: tumor necrosis factor-alpha, Ig-κ: immunoglobulin light chain kappa, Ig-λ: immunoglobulin light chain lambda.

Our analysis primarily focused on the linear regression analysis with the natural logarithm (LN) of the concentration of each studied uremic retention solute concentration as dependent variable and the CKD-EPI-Crea-CystC eGFR as independent variable. This formula was chosen as reference because it is considered as one of the most accurate ones at this time while it incorporates both Crea and CystC, in contrast to all other studied formulae which are based on either Crea or CystC [8]. Associations between eGFR and LMWP’s were expressed as regression coefficients (R^2^) and are summarized in table 3 and figure 1.

**Table 3:** Regression coefficients of LMWP’s and different eGFR formulae.

R^2^	CKD-EPI-Cr-CystC	MDRD	CKD-EPI-Cr	Stevens	Le Bricon	Rule
	**R^2^ >0.7**					
**Cyst C**	0.828	0.572	0.569	0.920	0.939	0.902
**β_2_M**	0.770	0.559	0.549	0.838	0.855	0.820
	**R^2^ 0.2–0.7**					
**RbP**	0.423	0.348	0.343	0.383	0.397	0.390
**Myoglobin**	0.303	0.246	0.262	0.287	0.297	0.293
**PTH**	0.231	0.130	0.132	0.279	0.274	0.276
	**R^2^ <0.2**					
**IL-6**	0.117	0.090	0.097	0.126	0.127	0.123
**Leptin**	0.084	0.056	0.059	0.092	0.065	0.065
**FGF-23**	0.058	0.008	0.008	0.095	0.101	0.094
**TNF-α**	0.056	0.043	0.044	0.066	0.058	0.061
**Ig-κ**	0.021	0.021	0.020	0.016	0.015	0.011
**Ig-λ**	0.000	0.000	0.000	0.001	0.001	0.001

LMWP: Low Molecular Weight Protein, R^2^: regression coefficient, eGFR: estimated glomerular filtration rate, CKD-EPI-Crea-CystC: CKD-EPI formula based on creatinine and cystatin C, MDRD: Modification of Diet in Renal Disease formula, CKD-EPI-Crea: CKD-EPI formula based on creatinine. CystC: cystatin C, β_2_M: beta-2-microglobulin, RbP: retinol binding protein, PTH: parathyroid hormone, IL-6: interleukin-6, FGF-23: fibroblast growth factor-23, TNF-α: tumor necrosis factor-alpha, Ig-κ: immunoglobulin light chain kappa, Ig-λ: immunoglobulin light chain lambda.

The R^2^-values per individual solute were divergent; according to these, associations could be arbitrarily divided into three groups: strong (R^2^ >0.7), moderate (R^2^ 0.2–0.7) and weak (R^2^ <0.2) (Figure 1). As expected, CystC (R^2^ = 0.828) was strongly associated as it is one of the used parameters in the formula. Only β_2_M showed a similar association (R^2^ = 0.770). Retinol binding protein (RbP), myoglobin and parathyroid hormone (PTH) were moderately associated to eGFR with R^2^-values of 0.423, 0.303 and 0.231, respectively. The association with eGFR was only weak for IL-6 (R^2^ = 0.117), leptin (R^2^ = 0.084), FGF-23 (R^2^ = 0.058) and TNF-α (R^2^ = 0.056). There was even no association for immunoglobulin light chain kappa (Ig-κ) (R^2^ = 0.021) and immunoglobulin light chain lambda (Ig-λ) (R^2^ = 0) (P = N.S.).

**Figure 2:**
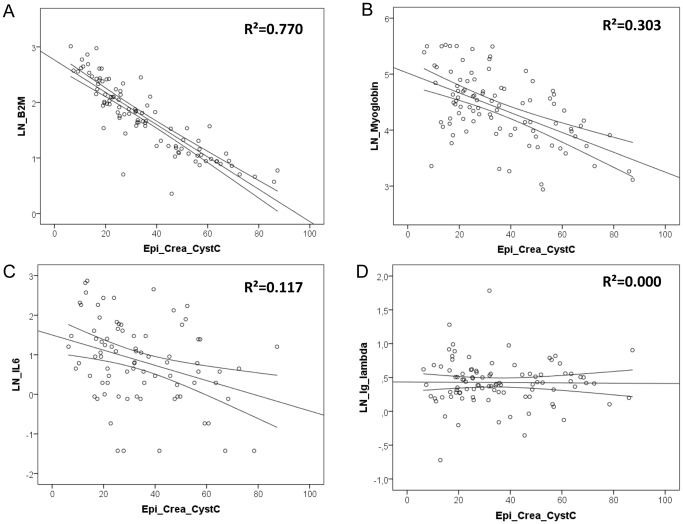
Dot plots with best fit linear regression lines for LN of LMWP’s in function of eGFR. Dot plots with best fit linear regression lines for natural logarithms of β_2_M, myoglobin, IL-6 and Ig-λ, as examples of strongly, moderately and weakly correlating low molecular weight proteins, in function of estimated Glomerular Filtration Rate, calculated by CKD-EPI-Crea-CystC. The dots represent the individual concentrations and the lines the best fit linear regression line with the 95% confidence interval. LN: natural logarithm, LMWP: low molecular weight protein, β_2_M : beta-2- microglobulin, IL-6: Interleukin-6, Ig-λ: immunoglobulin light chain lambda, EPI-Crea-CystC: CKD-EPI formula based on serum creatinine and Cystatin C, R^2^: regression coefficient, LN: natural logarithm.

Figure 2 shows the dot plots of solute concentrations as a function of eGFR. Whereas the relation is strong with little scatter around the linear regression line for β_2_M (Panel A), the scatter is much larger for solutes with moderate to weak R^2^-values, as illustrated for myoglobin (Panel B), IL-6 (Panel C) and especially Ig-λ (Panel D) for which there is no association at all. The large standard deviations or wide interquartile ranges of the individual solute concentrations per CKD-stage also illustrate the large inter-individual variability of LMWP concentration within the same eGFR-range (Table 2).

In addition we analyzed the correlation coefficients between the concentrations of the different LMWP and eGFR (CKD-EPI-Crea-CystC) in the group CKD stage 2–3 versus CKD stage 4–5. The correlation between RbP, PTH, myoglobin, FGF-23 and eGFR was significant in CKD-stage 2–3 while not in CKD stage 4–5. For the other investigated solutes, the R^2^-values in CKD stage 2–3 and CKD stage 4–5 were more confirm to each other. The respective R^2^-values are summarized in table 4.

**Table 4:** Regression coefficients (R^2^) of the concentration of LMWP’s and eGFR (CKD-EPI-Crea-CystC) comparing CKD stage 2–3 versus CKD stage 4–5.

R^2^	CKD stage 2–3(n = 48)	CKD stage 4–5(n = 47)
Cyst C	0.782	0.555
β_2_M	0.619	0.549
RbP*	0.254	0.001
PTH*	0.195	0.000
Myoglobin*	0.257	0.059
IL-6	0.074	0.117
TNF-α	0.107	0.087
Leptin	0.060	0.001
FGF-23*	0.120	0.033
Ig-κ	0.013	0.016
Ig-λ	0.001	0.006

LMWP: Low Molecular Weight Protein, eGFR: estimated glomerular filtration rate, CKD-EPI-Crea-CystC: CKD-EPI formula based on creatinine and cystatin C. CystC: cystatin C, β_2_M: beta-2-microglobulin, RbP: retinol binding protein, PTH: parathyroid hormone, IL-6: interleukin-6, TNF-α: tumor necrosis factor-alpha, FGF-23: fibroblast growth factor-23, Ig-κ: immunoglobulin light chain kappa, Ig-λ: immunoglobulin light chain lambda. *: LMWP’s with a significant correlation in CKD stage 2–3, but no significant correlation in CKD stage 4–5.

In a second step, the same analysis was performed with the other formulae under evaluation and compared to the results with the CKD-EPI-Crea-CystC-formula. The R^2^-values between all eGFR formula and individual solutes were strikingly similar with only one exception (Table 3). β_2_M was only moderately associated to Crea-based eGFR, with R^2^-values of approximately 0.55, as compared to CystC-based eGFR (R^2^ >0.8). In this way β_2_M followed the same pattern as CystC, for which this discrepancy could be attributed to whether CystC was included as a factor in the formula or not. Considering the other studied LMWP’s, only PTH showed a moderately similar trend, with R^2^ approximately 0.27 compared to R^2^ approximately 0.13, with CystC- or Crea- based eGFR-formulae, respectively.

Finally, we performed multifactorial regression analysis for the different LMWP’s with adjustment for several relevant variables. However, only two models induced a marked increase in R^2^-value: for leptin the association rose from weak to moderate when BMI was added to the regression model (R^2^ from 0.084 to 0.346), with BMI as an independent predictor for the leptin concentration. Likewise, after adjustment for CRP, IL-6 became moderately associated with eGFR (R^2^ from 0.117 to 0.305 after adjustment). For all other solutes there was no change in R^2^. (Data not shown).

## Discussion

We analyzed the linear regression coefficients between the concentrations of several LMWP’s retained in CKD and different eGFR-formulae in a CKD population, stage 2–5 not on dialysis. As a main finding, the R^2^-values diverged considerably, ranging from high, R^2^ >0.7, to low, R^2^ <0.2. The majority of the evaluated LMWP’s associated weakly (R^2^ <0.2 for IL-6, TNF-α, FGF-23 and leptin) or moderately (R^2^: 0.2–0.7 for RbP, myoglobin and PTH). There was no correlation at all for the immunoglobulin light chains. Only CystC and β_2_M showed a strong association with eGFR (R^2^ >0.7) (Figure 1, Table 3). Although in some studies a correlation was sought for individual LMWP’s and eGFR of mGFR, this present study seeked out the association of the concentration of several LMWP’s and eGFR formulae together allowing their comparison.

The R^2^-values for the weakly and moderately associating LMWP’s did not differ substantially whether eGFR was calculated with the CKD-EPI-Crea-CystC-formula [8], the Crea-based formulae (MDRD [7] and CKD-EPI-Crea [13]), or the three different CystC-based formulae, (Stevens [8], Rule [40] and Le Bricon [39]) (Table 3). These low regression coefficients can partially be attributed to the known limitations of eGFR, as an index of mGFR [6], [41]. However in at least four other studies, almost identical low regression coefficients were found between mGFR, assessed with different techniques, and the concentration of RbP (R^2^ 0.16) [42], myoglobin (R^2^ 0.38) [43], leptin (R^2^ 0.0004) [44] and FGF-23 (R^2^ 0.09) [45] as in our study, be it that transformation of the concentrations varied from study to study. In addition, the imperfect reflection of true GFR by eGFR can explain that regression coefficients are substantially lower than 1, but not that the range in between individual molecules is so discordant, whereas per molecule they are almost identical (Table 3, Figure 1). There was also an unpredictable and large variability in concentrations of different solutes within each eGFR stratum (Table 2). These data suggest another reason for the sometimes deceiving associations than a discrepancy among mGFR and eGFR, namely that uremic solute concentration depends on other factors than GFR as well. In this way, our study corroborates findings in an earlier study with small water-soluble and protein-bound compounds [15], [46].

These results are somewhat unexpected from a physiological point of view as the renal clearance of these LMWP’s depends to a large extent on GFR alone. All these LMWP’s are freely filtered through the GBM, followed, at normal physiological concentrations, by an almost entire uptake by the proximal tubules via a receptor-mediated process to be degraded subsequently into amino acids in the tubular lysosomes [17], [47]. In this way, the proximal tubulus plays an important role in LMWP metabolism but without a direct contribution to their renal clearance; regarding the latter, GFR is the rate limiting step. This probably explains why we did not find any association between eGFR and the total (free plus bound) immunoglobulin light chains, in contrast to Hutchison et al who evaluated only free light chains for their association to eGFR (free Ig-κ: R^2^: 0.52; free Ig-λ: R^2^: 0.44) [48], as only the free fraction passes the GBM. This is also in contrast to the small water-soluble and protein-bound uremic toxins, for which tubular secretion and/or reabsorption play an important role in renal clearance [15], [46].

However, the concentration of small water-soluble and protein-bound solutes may be further influenced by many other factors as well, such as enzymatic metabolism, intestinal secretion/absorption, generation by intestinal flora, diet and changes in distribution volume [15], [46]. It is conceivable that also the concentration of the weakly and moderately correlating LMWP’s depends on other mechanisms than GFR, which even seem to have more important weight than GFR. Some known influencing factors such as changes in generation, homeostatic mechanisms and extra-renal clearance are summarized in table 5. Multifactorial regression analysis for the respective LMWP’s including some of these parameters, increased the R^2^-value as expected. E.g. for leptin, R^2^ rose from 0.084 to 0.346 when corrected for BMI,which was an independent covariate for leptin concentration in a model with eGFR and BMI. The R^2^-value between IL-6 and eGFR became 0.305 instead of 0.117, when adjusted for CRP, which, in contrast to BMI for leptin concentration, did however not independently predict IL-6 concentration in a model with eGFR and CRP. The majority of potentially influencing factors did however not importantly affect the R^2^-values. This suggests that other than well known mediators may influence these LMWP concentrations as well. In more advanced CKD, the influence of confounders, for example bone metabolism, is probably more important, which could explain partially that no significant associations were observed between PTH or FGF-23 and eGFR in CKD stage 4–5, while they were present in CKD stage 2–3 (table 4). Another contributing factor to this discrepancy in associations between CKD stage 2–3 versus CKD stage 4–5 might be purely mathematical, as the GFR-range in CKD 2–3 (30–90 ml/min/1.73 m^2^) is much larger than CKD 4–5 (±10–30 ml/min/1.73 m^2^).

**Table 5:** Main factors influencing the concentrations of the studied LMWP’s, other than GFR.

	Extra renal handling	Generation
**CystC**	–	Gender, age, hyperthyroidism, corticosteroid intake, malignancy, inflammation
**β_2_M**	+ (∼5%)	Inflammation, malignancy
**RbP**	?	Insulin resistance, obesity, DM, Zn-deficiency, liver dysfunction, infection
**PTH**	+	Hypocalcemia, hyperphosphatemia, hypo-VitD
**Myoglobin**	+ (in uremia?)	Different generation in uremia (?)
**Leptin**	+	Obesity, gender, low energy expenditure, insulin resistance
**IL-6**	+	Inflammation
**TNF-α**	+	Inflammation
**FGF-23**	+ ?	Hyperphosphatemia, regulation mineral metabolism
**Ig-κ**	+ ?	B-cell lymphoproliferative disorders, inflammation
**Ig-λ**	+ ?	B-cell lymphoproliferative disorders, inflammation

CystC: Cystatin C, β_2_M: beta-2-microglobulin, RbP: retinol binding protein, PTH: parathyroid hormone, IL-6: interleukin-6, TNF-α: tumor necrosis factor-alpha, FGF-23: fibroblast growth factor-23, Ig-κ: immunoglobulin light chain kappa, Ig-λ: immunoglobulin light chain lambda, Zn: Zinc, DM: diabetes mellitus, Ca: Calcium, P: Phosphorus, VitD: Vitamin-D. For references: see Table S1.

This study demonstrates that eGFR is not a reliable predictor of the concentration of most of the evaluated LMWP’s, although several of them such as IL-6 [23]–[26], TNF-α [27], [28] and FGF-23 [31]–[33], [49] have been associated with mortality or with intermediate endpoints, such as vascular dysfunction or progression to end stage renal disease (ESRD) in CKD- or hemodialysis patients. Presumably, some of these solutes, especially if they would be representative for a cluster of other solutes, might by themselves become useful predictors of morbidity or mortality in CKD independently from eGFR. Based on the data collected in the present study, we investigated the mutual correlations between the concentrations of the different LMWP’s; however, we could not identify such a marker, correlating strongly to other LMWP’s without correlating to eGFR, among the investigated solutes (data not shown). This question, however, would be worthwhile to be investigated in larger populations.

In contrast to these weakly and moderately correlating LMWP’s, there is a remarkable similarity in regression coefficients between CystC and β_2_M. First, these molecules are the sole LMWP’s studied that result in acceptably high associations with eGFR (Table 3). Second, they associate better with CystC-based eGFR formulae [8], [39], [40] than with Crea-based ones [7], [13], the CKD-EPI-Crea-CystC [8] which contains both factors being intermediate (Table 3). Whereas this is no surprise for CystC which is included in some formulae and not in others, the pattern for β_2_M seems to be identical. This suggests that the kinetics of both molecules during progression of CKD depend on similar factors or at least factors with a similar impact on solute concentration. Of note, some of the non-renal elements with impact on both concentrations [50], [51], like chronic inflammatory disease or malignancy were among the exclusion criteria of this study. CystC was a superior marker of the association of GFR with outcome in a study by Peralta *et al*., who showed that the predictive value for mortality or CVD of eGFR <60 ml/min/1.73 m^2^, based on a CystC-based eGFR, was better than eGFR based on the CKD-EPI-Crea-formula [45]. Recently, in a general population, CystC and β_2_M were stronger predictors of mortality, CVD and evolution to ESRD than eGFR based on the CKD-EPI-Crea [52].

The present study has some shortcomings. First, the study population was rather small, with even smaller subgroups per CKD-stage. Second, we used eGFR which gives only an approximate value for glomerular filtration in comparison to more exact methods such as EDTA-clearance. We preferred to use methods which are applied on a day to day basis. As the differences in correlations are so striking, it is very likely that these findings can be extrapolated to GFR in general. The strengths of this study lie in the fact that several LMWP’s are evaluated together in the same population for different eGFR formulae based on Crea, CystC or both.

Our present data, together with the previous ones [15], showing extremely variable associations between uremic retention solutes and a surrogate of GFR, suggests that eGFR per se is an inadequate indicator of the uremic status. This is also suggested by other studies. In a CKD population, Lilitkarntakul *et al.* [53] demonstrated that renal function did not independently predict arterial stiffness or endothelial dysfunction while the uremic retention solutes asymmetric dimethylarginine (ADMA), isoprostanes or endothelin-A did. In the Initiating Dialysis Early and Late (IDEAL) trial [54], approximately 75% of the patients randomized to start dialysis at low eGFR (5–7 ml/min/1.73 m^2^), initiated dialysis earlier, mainly because of uremic symptoms.

In this study, the regression coefficients of different LMWP’s in relation to eGFR are diverse and in general low. This shows that other factors than GFR are important for the development of the ‘uremic status’. Further research is needed to evaluate whether these uremic toxins can be used as biomarkers for the risk stratification associated to uremic toxicity within the different CKD-stages and beyond eGFR.

## Supporting Information

Table S1 Main factors influencing the concentrations of the studied LMWP’s, other than GFR.
(PDF)
