# Supplementary material for: Estimated Glomerular Filtration Rate Is a Poor Predictor of the Concentration of Middle Molecular Weight Uremic Solutes in Chronic Kidney Disease
Source: PLoS One. 2012 Aug 31;7(8):e44201. doi: 10.1371/journal.pone.0044201 (PMC3432070; doi:10.1371/journal.pone.0044201)
Supplement: Table S1 — Main factors influencing the concentrations of the studied LMWP’s, other than GFR. (PDF) [file pone.0044201.s001.pdf]

SUPPORTING INFORMATION FILE

Table S1: Main factors influencing the concentrations of the studied LMWP's, other than GFR

	**Extra renal handling**	**Generation**	**Reviewed in**
**CystC**	-	Gender, age, hyperthyroidism, corticosteroid intake, malignancy, inflammation	[55]
**β_2M**	+ (~5%) [56]	Inflammation, malignancy	[57]
**RbP**	?	Insulin resistance, obesity, DM, Zn-deficiency, liver dysfunction, infection	[58–61]
**PTH**	+ [62,63]	Hypocalcemia, hyperphosphatemia, hypo-VitD	[64,65]
**Myoglobin**	+ (in uremia?) [56]	Different generation in uremia (?)	[66]
**Leptin**	+ [67,68]	Obesity, gender, low energy expenditure, insulin resistance	[69,70]
**IL-6**	+ [70–72]	Inflammation	[73]
**TNF-α**	+ [74]	Inflammation	[73,75]
**FGF-23**	+ ?	Hyperphosphatemia, regulation mineral metabolism	[65,76,77]
**Ig-κ**	+ ? [78]	B-cell lymphoproliferative disorders, inflammation	[79]
**Ig-λ**	+ ? [78]	B-cell lymphoproliferative disorders, inflammation	[79]

CystC: Cystatin C, β_2M: beta-2-microglobulin, RbP: retinol binding protein, PTH: parathyroid hormone, IL-6: interleukin-6, TNF-α: tumor necrosis factor-alpha, FGF-23: fibroblast growth factor-23, Ig-κ: immunoglobulin light chain kappa, Ig-λ: immunoglobulin light chain lambda, Zn: Zinc, DM: diabetes mellitus, Ca: Calcium, P: Phosphorus, VitD: Vitamin-D.

Reference List

55. Seronie-Vivien S, Delanaye P, Pieroni L, Mariat C, Froissart M, et al (2008) Cystatin C: current position and future prospects. Clin Chem Lab Med 46: 1664-1686.

56. Floege J, Wilks MF, Soose M, Kotzerke J, Shaldon S, et al (1990) Renal Elimination of Beta-2-Microglobulin and Myoglobin in Patients with Normal and Impaired Renal-Function. Nephron 55: 361-367.

57. Drueke TB, Massy ZA (2009) Beta2-microglobulin. Semin Dial 22: 378-380.

58. Redondo C, Burke BJ, Findlay JB (2006) The retinol-binding protein system: a potential paradigm for steroid-binding globulins? Horm Metab Res 38: 269-278.

59. Theodosiou M, Laudet V, Schubert M (2010) From carrot to clinic: an overview of the retinoic acid signaling pathway. Cell Mol Life Sci 67: 1423-1445.

60. Mody N, Graham TE, Tsuji Y, Yang Q, Kahn BB (2008) Decreased clearance of serum retinol-binding protein and elevated levels of transthyretin in insulin-resistant ob/ob mice. Am J Physiol Endocrinol Metab 294: E785-E793.

61. Kotnik P, Fischer-Posovszky P, Wabitsch M (2011) RBP4: a controversial adipokine. Eur J Endocrinol 165: 703-711.

62. Liao S, Qie JK, Xue M, Zhang ZQ, Liu KL,et al (2010) Metabolic stability of human parathyroid hormone peptide hPTH (1-34) in rat tissue homogenates: kinetics and products of proteolytic degradation. Amino Acids 38: 1595-1605.

63. Jones KO, Owusu-Ababio G, Vick AM, Khan MA (2006) Pharmacokinetics and hepatic extraction of recombinant human parathyroid hormone, hPTH (1–34), in rat, dog, and monkey. J Pharm Sci 95: 2499-2506.

64. Kumar R, Thompson JR (2011) The Regulation of Parathyroid Hormone Secretion and Synthesis. J Am Soc Nephrol 22: 216-224.

65. Komaba H, Fukagawa M (2010) FGF23–parathyroid interaction: implications in chronic kidney disease. Kidney Int 77: 292-298.

66. Hallgren R, Karlsson FA, Roxin LE, Venge P (1978) Myoglobin Turnover - Influence of Renal and Extra-Renal Factors. J Lab Clin Med 91: 246-254.

67. Cumin F, Baum HP, Levens N (1997) Mechanism of leptin removal from the circulation by the kidney. J Endocrinol 155: 577-585.

68. Garibotto G, Russo R, Franceschini R, Robaudo C, Saffioti S, et al (1998) Inter-organ leptin exchange in humans. Biochem Biophys Res Commun 247: 504-509.

69. Meier U, Gressner AM (2004) Endocrine Regulation of Energy Metabolism: Review of Pathobiochemical and Clinical Chemical Aspects of Leptin, Ghrelin, Adiponectin, and Resistin. Clin Chem 50: 1511-1525.

70. Power ML, Schulkin J (2008) Sex differences in fat storage, fat metabolism, and the health risks from obesity: possible evolutionary origins. Br J Nutr 99: 931-940.

71. Garibotto G, Sofia A, Balbi M, Procopio V, Villaggio B, Tarroni A, Di MM, Cappelli V, Gandolfo MT, Valli A, Verzola D (2007) Kidney and splanchnic handling of interleukin-6 in humans. Cytokine 37: 51-54.

72. Castell J, Klapproth J, Gross V, Walter E, Andus T, Snyers L, Content J, Heinrich PC (1990) Fate of interleukin-6 in the rat. Eur J Biochem 189: 113-118. Article.

73. Stenvinkel P, Ketteler M, Johnson RJ, Lindholm B, Pecoits-Filho R, Riella M, Heimburger O, Cederholm T, Girndt M (2005) IL-10, IL-6, and TNF-alpha: central factors in the altered cytokine network of uremia--the good, the bad, and the ugly. Kidney Int 67: 1216-1233.

74. Ferraiolo BL, McCabe J, Hollenbach S, Hultgren B, Pitti R, Wilking H (1989) Pharmacokinetics of recombinant human tumor necrosis factor-alpha in rats. Effects of size and number of doses and nephrectomy. Drug Metab Dispos 17: 369-372.

75. Schottelius AJG, Moldawer LL, Dinarello CA, Asadullah K, Sterry W, Edwards CK (2004) Biology of tumor necrosis factor-+¦ΓÇô implications for psoriasis. Exp Dermatol 13: 193-222. 10.1111/j.0906-6705.2004.00205.x.

76. Seiler S, Heine GH, Fliser D (2009) Clinical relevance of FGF-23 in chronic kidney disease. Kidney Int 76: S34-S42.

77. Liu S, Quarles LD (2007) How Fibroblast Growth Factor 23 Works. J Am Soc Nephrol 18: 1637-1647.

78. Epstein WV, Gulyassy PF, Tan M, Rae AI (1968) Effect of Renal Homotransplantation on the Metabolism of the Light Chains of Immunoglobulins. Ann Intern Med 68: 48-62. Article.

79. Cohen G, Horl WH (2009) Free immunoglobulin light chains as a risk factor in renal and extrarenal complications. Semin Dial 22: 369-372.
